# Assessing, Advising, and Advancing the Filling Practices of the Radiology Request Form in Africa: A Systematic Review

**DOI:** 10.3390/diagnostics14151694

**Published:** 2024-08-05

**Authors:** Mohamed Hajalamin, Almontasir Awadalla, Mahmoud Mukhtar

**Affiliations:** 1 St Mary’s Hospital Phoenix Park, D20 TY72 Dublin, Ireland; 2 Beaumont Hospital, Beaumont Road, Beaumont, D09 V2N0 Dublin, Ireland; awademontasir@gmail.com (A.A.); m7mod.a7med@hotmail.com (M.M.)

**Keywords:** radiology request form, Africa, RRF

## Abstract

Despite the increased use of diagnostic imaging in Africa, the completion of the Radiology Request Form (RRF) remains suboptimal, often relying on paper-based communication. To examine the practices surrounding RRF completion in the African continent, on 25 March 2024, we conducted a systematic review of peer-reviewed articles describing the practice in African settings. Non-African studies, studies involving non-human subjects, and articles examining the practice of the RRF for interventional usage were excluded. Our search involves PubMed/MEDLINE, ScienceDirect, Scopus, Web of Science, Google Scholar, and African Journals Online. The included studies were 3004, of which 30 met the inclusion criteria. These studies span eight countries and highlighted several shortcomings, including the usage of informal forms, unconventional abbreviations, illegibility, inaccuracy, and the lack of important fields from institutional forms, commonly the last menstrual period and the referrer’s contact details. We also found widespread non-compliance in all RRF fields; half of the studies did not have an adequately filled form. Incomplete RRFs lead to delayed imaging, increased workloads for radiographers and radiologists, and potential misdiagnoses due to insufficient information. It will also impede the application of radiation protection principles. To address these challenges, empowering radiographers and radiologists and encouraging best practices is essential. Regular audits and educational initiatives aimed at clinicians are recommended. While transitioning to a paperless communication system might help, implementing nationwide quality improvement projects to standardise radiology request forms is currently more feasible.

## 1. Introduction

Africa has used diagnostic imaging more extensively, which has had favourable outcomes in patient care. However, there is a risk that the negative consequences resulting from unjustified exams could outweigh these benefits [1]. Because of the expanding imaging service nowadays, medical practice has become the primary source of ionising radiation exposure, surpassing any other human activity [2]. Therefore, the principles of radiological protection, which include justification, optimisation, and application of dose limits, must govern the use of diagnostic radiology [2]. The responsibility of applying those principles is shared between the radiology personnel and the referring clinicians. Effective communication will ensure that the benefits outweigh the harm and the exam is optimised.

At the heart of this communication lies the Radiology Request Form (RRF), a vital tool for referring patients for radiological examinations. Despite its significance, the proper completion of the RRF is often overlooked [3]. Through the years, efforts have been made to raise awareness and address the issues of unjustified imaging exams; several initiatives like AFROSAFERAD, Technical Cooperation in Africa, and Global Initiative on Radiation Safety in Healthcare Settings have made forward steps and even started to establish national guidelines for clinical imaging referral [4]. However, those achievements will face the hazard of non-continuity if a central tool such as the RRF is not given the attention it deserves by healthcare practitioners. 

Globally, inadequate provision of the RRF has been a matter of concern. Studies conducted in Australia [5], Brazil [6], Greece [7], India [8], Italy [3], Nepal [9,10,11], Pakistan [12,13], Saudia Arabia [14,15], Sri Lanka [16], United Kingdom [17,18,19], and the United States [20,21,22,23] have all shown substandard practice in the provision of adequate RRFs. This reflects a worldwide challenge affecting various healthcare settings across diverse regions and extending to all imaging modalities. According to the Royal College of Radiology, the RRF must include sufficient clinical and demographic information that details the patient’s clinical context and the intended recipient of the report [24]. This issue was also emphasised by many radiological societies, e.g., the American College of Radiology [25], the European Society of Radiology [26], and the Royal Australian and New Zealand College of Radiologists [27]. 

This study aims to provide insights into the practice surrounding RRFs in Africa by systematically reviewing the published African literature. While this issue has been reported in many non-African countries, we chose to review the practices on our continent. We believe these issues are more relevant in areas with limited resources, making focusing on our local context important. To our knowledge, this is the first review of its kind on a continental level.

## 2. Material and Methods

This study is a systematic review following the recommended Preferred Reporting Items for Systematic Reviews and Meta-Analyses (PRISMA) [28]. This study was not registered prior. 

### 2.1. Literature Search

On 25 March 2024, we conducted a systematic and comprehensive literature search for peer-reviewed publications across multiple databases, including PubMed/MEDLINE, ScienceDirect, Scopus, Web of Science (WOS), and Google Scholar. We applied no language, year, or study design filters to the search. We employed the search terms “radiology request form”, “radiological request form”, and “imaging request forms” to assemble relevant articles. Additionally, we extended our search to the reference lists of eligible articles and conducted a manual search in PubMed, Google Scholar, and African Journals Online. A detailed search strategy is outlined in Table S1.

### 2.2. Inclusion and Exclusion Criteria

The inclusion criteria were (a) African study, (b) observational design, (c) report relevant data on RRF practice, and (d) studies intended for diagnostic use. The exclusion criteria were (a) overlapping datasets, (b) where only abstract is available, (c) animal or in vitro studies, (d) duplicated and irrelevant data, and (e) non-peer-reviewed articles.

### 2.3. Selection Process

Identified studies were imported into EndNote X9.3.3 (Clarivate, Philadelphia, PA, USA) and grouped to remove duplicates automatically and under supervision based on matching fields of title, authors, year of publication, and journal name. Furthermore, studies were imported to Microsoft Excel version 16.87 (Microsoft Corporation, Redmond, WA, USA) and underwent manual duplicate removal through alphabetical sorting. M.H. managed the search, importing, and duplicate removal while two reviewers (A.A. and M.K.) independently screened the titles and abstracts against the predetermined inclusion and exclusion criteria. Where possible, the full texts of included articles are retrieved and subjected to full-text screening by M.H., A.A., and M.M. to make a final inclusion decision. Any conflicts in screening were resolved through consensus. 

### 2.4. Data Extraction

After final inclusion, all authors independently extracted the following data: author name, country, number of evaluated RRF, percentages of adequately filled forms, and percentages of adequate completion of patient’s name, age, gender, LMP, hospital number, residential address, location, clinical information, provisional diagnosis, request exam, anatomical site, previous imaging studies, referrer’s name, referrer’s phone number, referrer’s signature, consultant name, the urgency of the exam, ambulatory status, date of the request, request number, status of Diabetes Mellitus, Creatinine or eGFR, and allergy. We also extracted whether issues of illegibility, inappropriate abbreviations, and informal forms were reported. Extracted data were then cross-checked, and any disparities were resolved through consensus. Our original data extraction sheet is accessible in the Supplementary (Data Extraction Sheet S1).

### 2.5. Quality Assessment

In this systematic review, we used the STROBE (Strengthening the Reporting of Observational Studies in Epidemiology) checklist [29] to assess the quality of the included observational studies. The checklist consists of 22 items that cover all aspects of observational research. Each study was evaluated against these criteria and categorised as adequate, inadequate, or no information for each item. The quality metrics were filled in an Excel sheet and then uploaded into the Robvis tool [30] to generate visual representations of the quality assessments, including traffic light and summary plots. The quality assessment was independently conducted by A.A. and M.M., with any conflicts resolved through discussions and the involvement of the main author, M.H.

### 2.6. Statistical Analysis

Given the limitations and variability of the studies, we did not conduct a meta-analysis; instead, we mentioned the descriptive statistics of the extracted data. For each field of RRF, the maximum, minimum, mode, and median were reported using Microsoft Excel version 16.87 (Microsoft Corporation, Redmond, WA, USA). We chose not to report the median if the dataset was small, i.e., less than five studies. We also documented the presence or absence of issues related to illegibility, unconventional abbreviations, and the use of informal forms. 

## 3. Results

### 3.1. Search Results

The initial electronic search across seven databases yielded 3004 studies. Following duplicate removal and title/abstract screening, 43 relevant studies underwent full-text screening. Of these, 24 studies were eligible.

**Figure 1:**
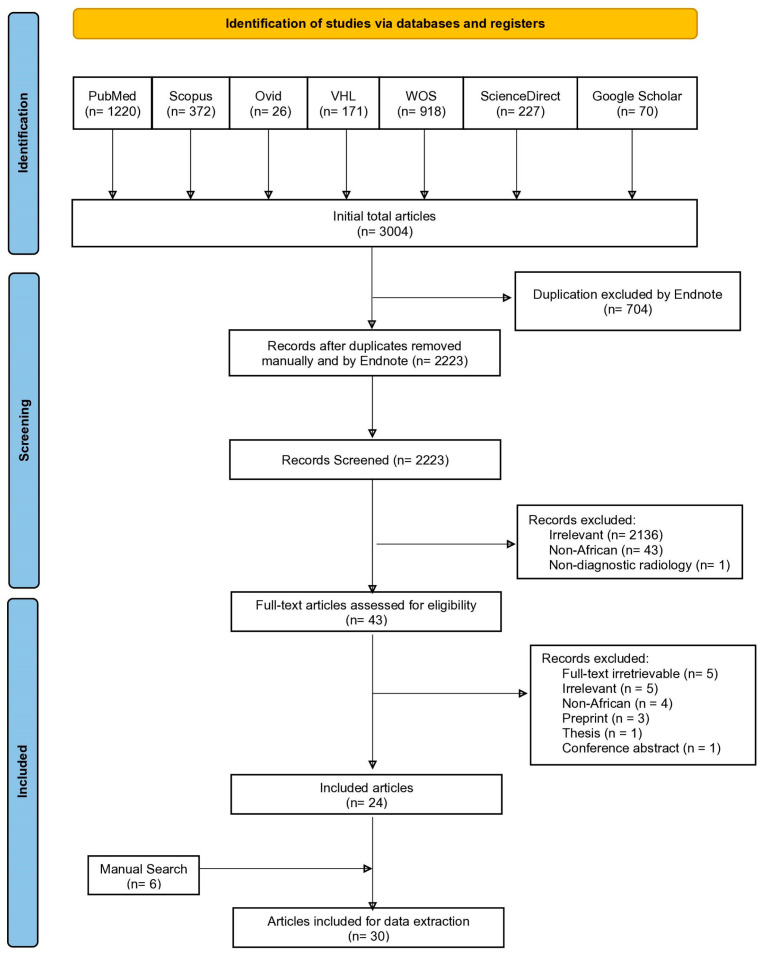
PRISMA flowchart demonstrating the inclusion process.

**Figure 2:**
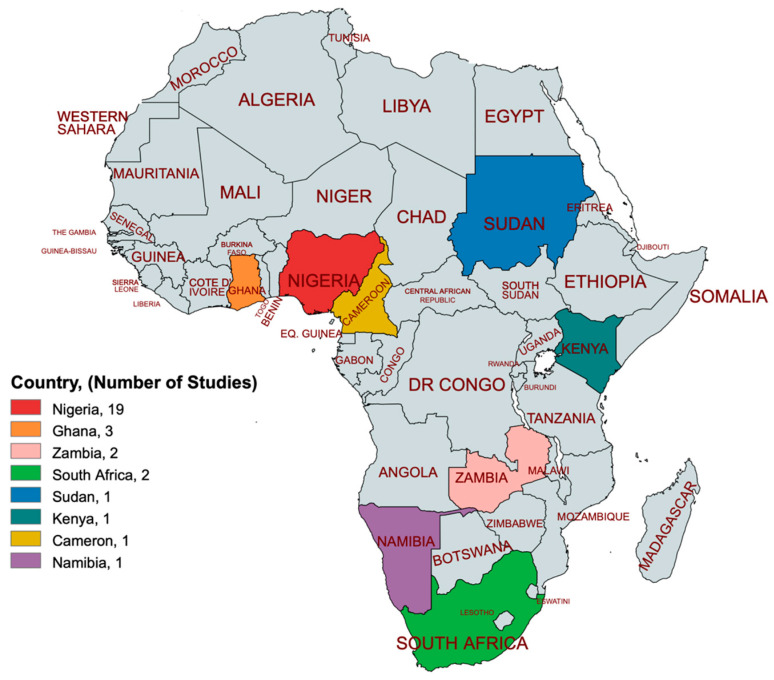
Countries of the studies created by mapchart.net.

The manual search yielded six relevant studies. In total, 30 eligible studies were included in the synthesis [31,32,33,34,35,36,37,38,39,40,41,42,43,44,45,46,47,48,49,50,51,52,53,54,55,56,57,58,59,60], as illustrated in the PRISMA flow chart (Figure 1). The eligible studies span from 2009 to 2023, representing eight countries: Nigeria, Ghana, Zambia, South Africa, Sudan, Kenya, Namibia, and Cameroon, as shown in Figure 2.

### 3.2. General Characteristics of the Studies

The number of reviewed RRFs ranged from 80 to 4465, involving various modalities, including plain X-ray, Computed Tomography (CT), Magnetic Resonance Imaging (MRI), Ultrasound (U/S), mammography, and hysterosalpingography. The majority of the healthcare facilities were teaching hospitals. It was often unclear what the standard was—whether it adhered to local or global guidelines or literature. Except for two facilities [38,42], all used paper forms. In Table 1, we detail the general characteristics of the included studies.

### 3.3. Quality Assessment

**Figure 3:**
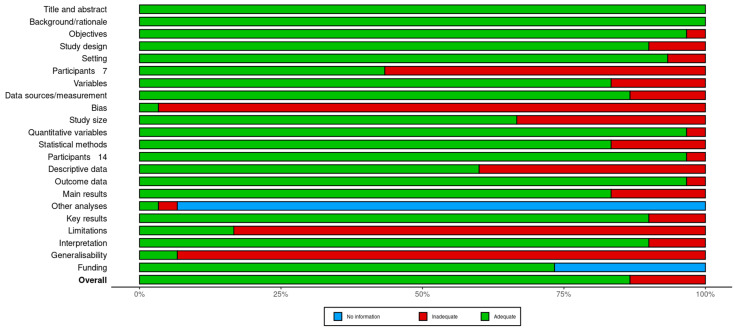
Summary plot for quality assessment.

As shown in Figure 3, generalisability and addressing bias were the primary concerns. Numerous studies failed to address the applicability of their findings to other settings or populations, and only a limited number discussed potential bias sources or their mitigation strategies. However, most of the studies demonstrated satisfactory adherence to several STROBE guidelines. A detailed assessment of each study is provided in Figure S1.

### 3.4. Completion Practice per Fields

In Table 2, we classified the information in the RRF into five categories and reported the number of evaluating studies and the descriptive statistics of completion rates of each field. The completion rates of all fields in all studies are reported in Table S3. It is noted that researchers tended to focus on evaluating some RRF components and ignore others. Fields like clinical information, patient’s name, and age are consistently reviewed, but others, such as the urgency of the exam, exam date, and last menstrual period (LMP), are often neglected.

Patient’s biodata

Name

We found that 25 studies evaluated the completion rate of the name, which ranged from 70.2% to 100%. Most of these studies reported near-full completion, with full compliance observed in 15 studies. The median and mode values were 100%, indicating a high level of adherence. This field was the only parameter that achieved a median of 100%.

Age and Date of Birth

Our analysis reveals a wide variation in age completion, with full completion observed only in one study [38]. The median value was 86%, suggesting higher compliance in general. However, the minimum was 20.57%. 

Gender and LMP

In our review, 21 studies revealed completion rates ranging from 74% to 100%. Approximately 75% of these studies reported gender completion rates exceeding 94%, demonstrating generally high compliance. In contrast, the completion rate of LMP was notably poor. It was only investigated in 11 studies, and ranged widely, from as low as 0.9% to 62.32%. 

Address

It was evaluated in 16 studies, with completion rates ranging from 2.1% to 65.3%. The results generally indicate poor compliance, with several studies reporting completion rates below 10%, and more than half had completion rates below 50%.

Hospital Number

The completion of the hospital number was investigated in 15 studies and showed the greatest variability, ranging from none to full completion. While some studies report relatively high completion rates, such as 80.40%, 86.55%, 87.1%, 92.3%, 92.7%, and 93.7%, many others indicate much lower completion rates, including 2.7% and 10%.

Location and ambulatory status

Nineteen studies evaluated the patient’s location, revealing a considered variability in completion rates from 35% to 100%. With many studies reporting rates above 90%, others recorded moderate rates, like 64.6%, 72.4%, and 75%. Oppositely, only eight studies examined the patient’s ambulatory status, making it one of the least studied fields. Despite higher completion rates in the patient location field, ambulatory status generally showed lower completion rates, with most studies below 20%. 

Clinical information and the clinical question

The median completion rate for clinical information is 82.8%, indicating an average but wide variability ranging from 18.2% to 99.4%. On the other hand, the clinical question was the fourth least examined field and was only investigated in 10 studies, with completion rates ranging from 23.7% to 98.4% and a median of 67%. 

Requested exam

As anticipated, most studies report high completion rates for requested exams, with many achieving near or full completion. A few studies report completion rates slightly below 90% [31,39,54,58]. Despite this, the median completion rate across studies remains high at 99.45%. 

Anatomical site

The anatomical site was the second least examined field, with only five evaluating studies. Two studies had near-full and full completion [44,46], while the remaining did not exceed 93.1%, with a minimum of 42.6%.

Previous imaging studies

Fifteen studies evaluated the completion of previous radiological exams, and the rates ranged from 0% to 69%. The median and mode rates were 7.4% and 0.0%. 

Previous surgical history

Eight studies assessed the completion rates of previous surgeries and exhibited considerable variability, ranging from 0.35% to 59.8%.

Referrer’s contacts

The completion rates for the referrer’s name were examined in 23 studies, varying significantly from 2.3% to 99%. In contrast, the referrer’s phone number was the second least examined field, evaluated only in seven studies, with many reporting none or very low completion rates, making it amongst the most poorly filled fields. 

Consultant in charge

Many studies report high completion rates for the consultant’s name, with several studies achieving rates exceeding 90% and generally maintaining above 70%. The median stands at 83.17%**.**

Urgency

Two studies examined the completion rates, which were 3.9% and 63%. It was the least examined field by the researchers. The scarcity of data will not allow for the drawing of interpretable conclusions.

Date of request

Generally, there are high completion rates for the date of the request, with rates ranging from 75% to full completion. 

Diabetes, allergy, and kidney function

Only four studies reported allergies to radiopharmaceutical agents, each with extremely low completion rates below 8% [34,36,41,48]. Similarly, one study mentioned information on diabetic status and kidney function, which noted a poor completion rate [36].

## 4. Discussion

### 4.1. Outlines of the Current Practice

To our knowledge, this is the first paper to review the practice surrounding the RRF on a continental level. It aimed to shed light on the practice within Africa by systematically reviewing the published African literature. We found that inadequate provision of the RRF is a global issue [3,5,6,7,8,9,10,11,12,13,14,15,16,17,18,19,20,21]. This pitfall might eventually result in inaccurate diagnostic conclusions, leading to inappropriate interventions and unnecessary exposure for patients and healthcare providers [32]. Therefore, accurate and adequate filling of the RRF cannot be overstated, especially in low-resource settings [31]. Garba et al. found that 20.9% of the examinations had been repeated due to missing or inappropriate information [39], which is an avoidable and unnecessary exposure.

In this review, we found variable degrees of non-compliance existed across all fields of the RRF. Fields such as the patient’s name, gender, and the requested exam show high completion rates, often reaching close to or at 100%. Meanwhile, the completion rates in other fields range from low to moderate. Of the 22 studies that reported the overall completion percentage of the RRFs, 14 found that none were fully completed, similar to a study in Pakistan [12]. Apart from this, several shortcomings were also noted: inaccurate filling, inappropriate use of abbreviations, illegibility, and the omission of critical parts from the formal RRF. In Table S3, we summarised important issues highlighted by the researchers in the studies.

While this paper does not focus on the appropriateness of the requested exams, four studies [31,33,37,38] highlighted that a significant number of requests were inappropriate (36%, 34.6%, 13.9%, and 9.2%), which, on the other hand, emphasises the importance of adequate filling of the RRF to allow the assessment of appropriateness. A study in Greece has found a similar degree of inappropriateness (41.2%) [7].

### 4.2. RRF Layout

Regarding the RRF layout and formality, we observed that some facilities received informal RRFs written on improvised plain sheets [37], drug prescriptions [36,47], laboratory forms, and continuation sheets [50]. Incorrect form usage, such as employing an X-ray request form for CT exams, was also noted [36]. The usage of unconventional forms is unsurprisingly prevalent in paper-based communication settings. A non-African study encountered this too [12]. The unavailability of standardised forms allows for omitting important information. The extent of informal form usage could be more significant than observed because nearly a third of the studies excluded them from the analysis [34,37,41,44,46,47,48,56]. 

Another fundamental problem was the design limitation and omission of critical parts from the institutional RRF, such as date of birth [36,47], gender [47], LMP [31,32,36,40,48], referrer’s contact details [31,47,48,60], referrer’s signature [47], previous imaging and surgeries [32,36,48], patient’s address [36], hospital number [40,47,48], clinical question [40], and ambulatory status [48]. In Table 3, we propose a categorised list that should be included in all radiology request forms to ensure adequate and effective documentation.

Additional concerns were the illegibility and inappropriate abbreviations, which can impede effective communication. Garba et al. reported that illegibility issues necessitated the repetition of 15% of plain X-rays [39]. In another study, a third of the requests, which is a massive number, were illegible and unclear [35]. Overall, ten studies found problems with legibility [31,35,36,39,41,50,51,56,58,59], and seven studies noted the use of unconventional abbreviations [35,41,48,52,56,58,60], which had been prevalent in more than half of the requests in the study of Jimah et al. [40]. However, this shortcoming had been widely reported outside Africa [8,10,12,16,20] with instances of good compliance like a study in Nepal [9]. Clarity of communication is mandatory in healthcare practices and should be tied to the highest possible standards. 

### 4.3. Shortcomings in RRF Completion

Moving on to the next point, we noticed that overall, the name, age, and the requested exam are often filled out adequately in line with studies outside Africa [9,11,12] yet contrasting a study in India [8]. The name is the patient’s first identifier, and it must be legible and stated in full, i.e., first name and last name. Age is an essential identifier and has clinical and procedural relevance, too. Its omission from radiology request forms can lead to avoidable challenges in clinical decision making [39,44]. Age and its full version, the date of birth (DOB), are essential for accurate patient identification and can greatly benefit disease differentiation. However, none of the studies assessed the completeness of DOB, possibly due to its substitution with the age field, as suggested by two authors [36,47]. Another recorded issue is stating the age as a general category (adults or children) instead of specifying the number [41,52,56,60]. 

Gender is another identifier and is particularly relevant because some diseases are gender-specific [44]. The compliance in our study was relatively high, with a median of 96%. Conversely, despite the known risks associated with radiation exposure during pregnancy [61], the completion rate of LMP was notably poor, agreeing with a study in Pakistan [12]. It is, however, interesting that requests for procedures such as hysterosalpingography lacked LMP [44]. We also found that LMP is absent from some institutional forms [13,14]. 

Among the patient identifiers, the hospital number is unique and especially important when patients cannot communicate or when similar information exists [35]. The residential address is also an important identifier and is necessary for contacting and tracing patients [41]. Both were poorly reported in most of the reviewed studies. A reason for that could be the paper-based settings where the requesting method is not linked to a previously stored database and the patient registration shortfalls in Africa. 

Regarding the location and ambulatory status, the ambulatory status was poorly examined and reported compared to the location. Stating the patient’s location enhances the accuracy of patient identification and can help prioritise examinations, as certain wards or settings may have higher rates of frailty and fatality [47]. Along with the patient’s mobility status, stating the location will also help the radiographer tailor the imaging technique accordingly [44]. Better results have been reported outside Africa [8,12].

When it comes to clinical information, in an updated systematic review by Castillo et al., well-provided clinical information was found to enhance the accuracy of interpretation, clinical relevance, and confidence in reporting [62]. Ahmed et al. found a significant correlation between inappropriate requests for ionising radiation tests compared to non-ionising [33]. This highlights the importance of optimum and accurate filling of clinical information to enable radiographers and radiologists to assess appropriateness and minimise the risk of unnecessary radiation exposure. In our study, the adequate completion rate ranged widely between 18.2% and 99.4%. Doctors should bear in mind that diagnostic radiologists, unlike referring clinicians, typically do not interact with patients and are not as familiar with them; thus, structuring the clinical information by mentioning the key findings in history, examination, and investigations besides stating a clinical question or provisional diagnosis is mandatory. The clinical questions have been much less evaluated and reported in our study compared to clinical information. Overall, there seems to be a trend where higher completion rates of clinical information correspond to higher completion rates of clinical questions and vice versa. However, this relationship is not absolute. It is worth highlighting that the main goal of diagnostic radiology is to address clinical inquiries through imaging techniques and assist in optimising care, which may involve the decision to refrain from conducting certain radiological tests [63]. The rationale for stating the clinical question is here. Globally, there is well-based work to improve RRFs by national standardisation of RRFs [15] and by specifying and tailoring the needed clinical information for different medical departments through Delphi studies [3,5]. Insufficient clinical information is an ongoing issue and is widely reported around the globe [8,10,11,12,13,16,18,20,21,22,23]. 

For the requested exam and the anatomical site, the requested exam was well reported as expected, but the anatomical site was reported in only five studies compared to 21 for the requested exam. It is important to accurately and legibly state the requested exam to prevent delay and unnecessary repetition. Garba et al. [39] found that 24% of radiological exams were repeated due to a lack of clarity regarding the requested exam, which is apparently unnecessary radiation, waiting time, and workload. The urgency of requests was the field that was examined the least, with only two evaluating studies, despite its central role in prioritising exams.

This review also showed that the past medical history involving surgeries and previous imaging studies has been evaluated in 8 and 15 studies with generally poor completion rates, pointing to widespread non-compliance. Similarly, the adequate completion of previous radiological studies was noted to be 23.4% in a study in Greece [7]. Stating past surgical and medical history is important to avoid misinterpretations related to anatomical distortions.

Reviewing the referrer’s details, we found very poor practice, especially in recording the phone or bleep numbers, which was the second worst parameter echoing results in Pakistan [12,13], Nepal [9,11], and the U.K. [17]. The reasons behind this may be the availability of alternative communication methods, limitations in the design of the RRFs, or a general lack of awareness. RRFs should contain complete referrer contact information to facilitate two-way communication and must also be signed to ensure medicolegal validation. 

Consistent with findings outside Africa [12,13], we observed most forms lacked information regarding patients’ diabetic and allergic statuses and kidney function—even in studies involving CT scans or other contrast-enhanced exams. They also lacked information about medical devices or implants, similar to a study in Brazil [6].

### 4.4. Potential Drivers of Poor Compliance

Africa has inefficient health administration and management systems that face challenges on multiple levels [64]. In such a setting, quality and safety in imaging are more relevant to maintaining and improving gains from the increased imaging use [1]. Before addressing the possible obstacles to adequate provision of RRFs, it is important to note that, due to the shortage of radiologists in Africa, imaging interpretations are sometimes performed by the requesting physician [65,66] or radiographers [66]. This can result in these practitioners neglecting to fill out the forms adequately or not filling them out at all.

However, fundamental and core barriers play a role in the shortcomings noticed in our study. For example, the high turnover [67] of radiology and radiography personnel makes it challenging to sustain good practices over time. The lack of consistent policies and guidelines to organise radiation exposure at the level of the continent will make local professional bodies less adherent to the standards. The financial and organisational infrastructure challenges impede the transition to an electronic health system [68]; as a result, issues like illegibility, non-compliance, and inappropriate abbreviation become inevitable with paper-based communication and are found to be significantly addressed following the transition into an electronic-based RRF [13,14]. 

On the other hand, some addressable issues do exist. For example, it was unclear what the local standard was for filling the RRF, which made it difficult to audit and identify the gaps in local practice. The higher prevalence of defensive medicine practice in Africa [69] will lead practitioners to follow the norm rather than evidence at the expense of radiation safety. The poor awareness of radiation exposure risks [70] and the paucity of proper training led professionals to be naive to the efficient and safe use of diagnostic radiology, which in turn ended in overuse or underuse [67]. The design limitations of the RRF and the omission of important parts will imply the unimportance of some information, which is contrary to the truth. 

The culture of medical dominance in the cycle of radiology acquisition has also been found to hinder the vigilant role of radiographers [71]. However, radiographers are the final point of contact before patients are exposed to radiation. They must advocate for patients, ensuring protection from unnecessary exposure, as this is their legal, ethical, and professional obligation [72].

To alleviate this situation, joint efforts between local institutions, professional bodies, and radiology departments should be made to empower radiographers and radiologists and promote a sense of professional accountability among medical practitioners. 

### 4.5. Study Limitation

While this study provides insights into the practices surrounding RRF completion in Africa, several limitations must be considered. Our review is based on data from only eight African countries, which may not fully represent the entire continent. The variability in evaluated modalities, sample sizes, and healthcare settings could affect generalisability. 

We noticed that the researchers tended to focus on evaluating some RRF components and ignoring the others. Fields like clinical information, patient’s name, and age are consistently reviewed, but others, such as the urgency of the exam, exam date, and LMP, are often neglected. Also, as revealed by the quality assessment, there are concerns about generalisability and managing bias. Therefore, an accurate picture is still to be obtained. 

Finally, our review is the first of its kind, laying the groundwork for future research. By conducting similar reviews on other continents, we can enhance the contextualisation of our findings, contributing at the end to a more inclusive understanding of the subject globally.

## 5. Conclusions

Negligence in completing radiology request forms is prevalent across Africa and mandates being addressed. A variable degree of non-compliance was observed across all fields. Radiographers, radiologists, and institutions could create ample room for impactful changes based on the ALARA principle, value-based healthcare, and evidence-based medicine principles. Effective communication in the patient’s best interests requires a thorough, clear, and complete message.

## 6. Recommendations and Future Directions

1. Radiographers should exercise their authority to return any RRFs that are inadequately filled, including those that are improvised.
2. Local radiological departments should conduct educational meetings highlighting the clinical and medicolegal importance of adequate RRFs, particularly targeting junior radiographers and doctors, along with regular local audits to maintain adherence to the best available practices.
3. Implement nationwide projects to unify and standardise a national RRF.
4. Local institutions should establish policies and guidelines to govern the use of diagnostic radiology services and their aiding tools, especially the radiology request form.
5. Integrate Radiation Protection Acts and regulations into medical school curricula and emphasise their mandatory application in clinical practice.
6. While transitioning to a paperless system could significantly address current issues, developing a well-designed, institutional RRF is a more immediately feasible and cost-effective solution.
7. We encourage African researchers to conduct studies evaluating RRF practice in their countries to draw a clearer picture of African problems and solutions from an African point of view.
8. Future studies should evaluate all fields of the RRF, especially those that were least examined in this review, such as LMP, clinical questions, ambulatory status, anatomical site, past medical history, referrer’s contact details, and exam urgency.
9. We finally recommend conducting similar reviews on continental levels to draw actual contextualisation and global understanding.

## Figures and Tables

**Table 1:** General characteristics of the studies.

Reference	Author, Year of Publication	Country	RRF Numbers	Modality	Type of Healthcare Facility	RRF Format	Percentage of Completely Filled Forms
[31]	Ademola et al., 2023	Nigeria	96	CT Chest	Public teaching hospital	Formal	None
[32]	Bwalya et al., 2023	Zambia	974	Plain radiography	Tertiary hospital	Formal	9.50%
[33]	Ahmed et al., 2022	Kenya	1053	X-ray, CT, U/S, and MRI	Public hospital	Formal	-
[34]	Chi et al., 2022	Nigeria	303	CT	University teaching hospital	Mixed *	None
[35]	Kuvare et al., 2022	Namibia	172	Pelvic and cervical spine plain X-ray	Public hospitals	Formal	6.6%
[36]	Chanda et al., 2021	Zambia	80	CT	Public hospital	Mixed	None
[37]	Donald et al., 2021	Nigeria	2053	X-ray, U/S, CT	Public tertiary hospital	Mixed	-
[38]	Edzie et al., 2021	Ghana	527	Obstetric sonography	Public tertiary hospital	Formal ^E^	-
[39]	Garba et al., 2021	Nigeria	158	Plain X-ray	Tertiary hospital	Formal	None
[40]	Jimah 2021	Ghana	500	CT, general and vascular U/S, hysterosalpingography, mammography, and other special studies	Public tertiary hospital	Formal	None
[41]	Robinson et al., 2021	Nigeria	1131	U/S, X-ray, CT, and MRI	Public tertiary hospital	Formal	-
[42]	Beviss-Challinor et al., 2020	South Africa	139	CT abdomen	Public tertiary hospital	Formal ^E^	None
[43]	A. Alameen et al., 2019	Sudan	216	-	Public tertiary hospital	Formal	None
[44]	Chukwuma et al., 2019	Nigeria	700	-	Public teaching hospital	Formal	24.8%
[45]	Khadija et al., 2019	Nigeria	400	U/S, CT, and plain X-ray	Tertiary hospital	Formal	0.98%
[46]	Mohammed et al., 2018	Nigeria	163	X-ray and CT	Teaching hospital	Formal	42.9%
[47]	Hk 2018	Ghana	189	X-ray	Primary public hospital	Formal	None
[48]	Onwuchekwa et al., 2017	Nigeria		X-ray, U/S, MRI, CT	Multicentre (three public hospitals)	Formal	None
[49]	Agi et al., 2015	Nigeria	2053	-	Public teaching hospital	Formal	-
[50]	Akintomide et al., 2015	Nigeria	580	Plain X-ray	Public tertiary teaching hospital	Mixed	None
[51]	Abubakar et al., 2015	Nigeria	339	Plain X-ray	Tertiary teaching hospital	Formal	None
[52]	Oyedeji et al., 2015	Nigeria	4465	-	Private tertiary diagnostic centre	-	≤1.3%
[53]	Danfulani et al., 2015	Nigeria	214	Plain X-ray	Public tertiary health centre	Formal	2.3%
[54]	Moifo et al., 2014	Cameron	262	U/S, CT, and plain radiography	Public university hospital	Formal	1.1%
[55]	Schouwenburg et al., 2014	South Africa	516	MRI	Public tertiary hospital	-	-
[56]	Afolabi et al., 2012	Nigeria	202	Plain and contrast studies X-ray	Public specialist hospital	Mixed	89.1%
[57]	Irurhe et al., 2012	Nigeria	300	Plain X-ray, CT, and U/S	Public teaching hospital	Formal	None
[58]	Yousef et al., 2011	Sudan	350	X-ray	Multicentre (four governmental teaching hospitals and one private specialised hospital)	Mixed	None
[59]	Adebayo et al., 2009	Nigeria	600	Plain radiography, U/S	Multicentre (three public teaching tertiary hospitals)	Formal	4.8%
[60]	Akinola et al., 2009	Nigeria	145	CT and MRI	Tertiary teaching hospital	Formal	None

* means the facility received formal and informal RRF. ^E^ stands for electronic RRF.

**Table 2:** Completion rates as per fields.

	Field (Number of Evaluating Studies)	Maximum %	Minimum %	Median %	Mode %, (Number of Frequencies)
Patient’s biodata	Name (25)	100%	70.20%	100%	100% (13)
	Age (25)	100%	20.57%	86%	None
	Gender (21)	100%	74%	96%	None
	LMP (11)	62.32%	0.9%	18.4%	18.4% (2)
	Address (16)	65.3%	2.1%	12.65%	None
	Hospital No (15)	100%	0.0%	71.4%	None
	Location (19)	100%	35.1%	86.3%	92.4% (2)
	Ambulatory status (8)	57%	1.4%	3.8%	1.4% (2)
	D.M. (1)	-	-	-	-
	Creatinine (1)	-	-	-	-
	Allergy (4)	8%	2.5%	-	None
Clinical data	Clinical information (29)	99.4%	18.2%	82.35%	98% (2)
	Clinical diagnosis or question to be answered (10)	98.4%	23.7%	66.6%	None
	Anatomical site (5)	100%	42.6%	93.1%	None
Radiological exam data	Requested exam (21)	100%	60%	99.45%	100% (6)
	Urgency (2)	63%	3.9%	-	None
	Date (19)	100%	75.5%	92%	97% (2)
Medical background	Previous imaging studies (15)	69%	0.0%	7.4%	0.0% (2)
	Previous surgeries (8)	59.8%	0.35%	10.4%	None
Referrer’s data	Name (23)	99%	2.3%	86%	None
	Phone or bleep number (7)	10%	0.0%	0.0%	0.00% (4)
	Signature (19)	100%	44%	89.3%	None
	Consultant name (14)	99.7%	56.4%	82.2%	None

**Table 3:** Proposed components to be included in the Radiology Request Form.

**Biodata**	Name
	Date of birth (DD/MM/YYYY)
	Gender
	LMP if child-bearing-age female
	Residential address
	Hospital number
	Ambulatory status
**Clinical information**	Clinical information
	Clinical question or provisional diagnosis
	Previous medical and surgical histories
	Previous imaging studies
	Location/department
**Requested exam information**	Request exam
	Anatomical site
	Urgency
	Date of the exam
	Allergies, eGFR or creatinine levels, and diabetic status for contrast studies
	Medical devices or safety checklist for MRI
**Referrer’s information**	Referrer’s name
	Referrer’s mobile/bleep number
	Referrer’s signature
	Name of consultant in charge

## Data Availability

All data are available within the manuscript and its Supplementary Material.

## Supplementary Materials

The following supporting information can be downloaded at: https://www.mdpi.com/article/10.3390/diagnostics14151694/s1, Table S1: Detailed search strategy; Figure S1: Quality assessment per study; Table S2: Completion rates of RRF fields; Table S3: Issues highlighted by researchers; Data Extraction Sheet S1.
