# Supplementary material for: Assessing, Advising, and Advancing the Filling Practices of the Radiology Request Form in Africa: A Systematic Review
Source: Diagnostics (Basel). 2024 Aug 5;14(15):1694. doi: 10.3390/diagnostics14151694 (PMC11311308; doi:10.3390/diagnostics14151694)
Supplement: Supplementary file 1 [file diagnostics-14-01694-s001.zip › Supplementary.pdf]

Table S 1 Search Strategy

Database	Search Term	Search within	Results
**PubMed**	((radiology request form) OR (radiological request form)) OR (imaging request forms)	All fields	1220
**Scopus**	((radiology request form) OR (radiological request form)) OR (imaging request forms)	Title, abstract and keywords	372
**Ovid**	((radiology request form) OR (radiological request form)) OR (imaging request forms)	All resources. Keywords	26
**VHL**	((radiology request form) OR (radiological request form)) OR (imaging request forms)	Title, abstract and subject	171
**Web of Science**	((radiology request form) OR (radiological request form)) OR (imaging request forms)	All fields	918
**Google Scholar**	allintitle: ((radiology request form) OR (radiological request form)) OR (imaging request forms)	Title	70
**ScienceDirect**	((radiology request form) OR (radiological request form)) OR (imaging request forms)	Title, abstract and keywords	227
**Total**	**3004**		

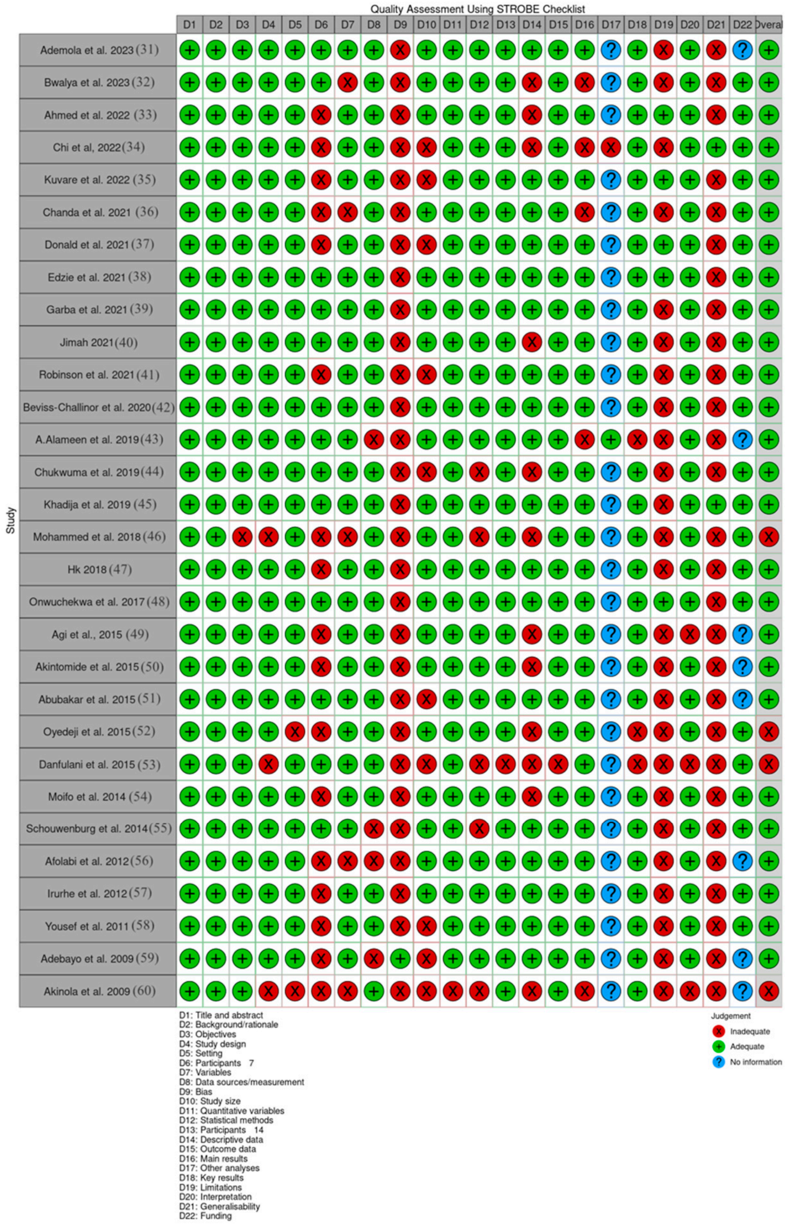

Figure S1 Quality Assessment Per Study

Table S 2 Completion rates of RRF fields

Reference	***Author, Year***	***Country***	***Fields***																											***Layout***		
			Patient name	Patient age	DOB	Patient gender	Patient address	LMP	Hospital Number	Patient location	Clinical information	Clinical question	Requested exam	Anatomical site	Previous exam	Previous surgeries	Referrer's name	Referrer's phone number	Referrer's signature	Consultant's name	Radiographer Name	Radiographer signature	Urgency	Ambulatory status	Date of request	Request number	Diabetes status	Creatinine levels	Allergy	Illegibility	Inappropriate abbreviation	Usage of informal form
(31)	Ademola et al. 2023	Nigeria	70.20%	57.40%	-	98.90%	-	-	57.40%	35.10%	46.20%	30.90%	60.00%	42.60%	3.20%	12.80%	47.90%	-	50.00%	56.40%	-	-	-	-	75.50%	-	-	-	-	Yes	-	-
(32)	Bwalya et al. 2023	Zambia	99.50%	99.70%	-	99.60%	-	-	39.7%	86.10%	53.80%	93.0%	99.60%	-	-	-	82.20%	-	96.80%	-	-	-	63.00%	-	-	-	-	-	-	-	-	-
(33)	Ahmed et al. 2022	Kenya	-	-	-	-	-	18.4%	-	-	81.30%	-	-	-	-	-	-	-	-	-	-	-	-	-	-	-	-	-	-	-	-	-
(34)	Chi et al, 2022	Nigeria	100%	94.70%	-	94.10%	65.30%	8.1%	92.70%	100%	84.50%	67.0%	100%	-	2.60%	-	89.90%	0.00%	89.90%	90.80%	-	0.00%	-	-	95.70%	99.70%	-	-	5.90%	-	-	Yes
(35)	Kuvare et al. 2022	Namibia	99.00%	87.00%	-	98.40%	-	-	35.00%	87.00%	99.00%	98.40%	100%	-	-	-	83.00%	-	98.00%	-	-	16.10%	-	57.00%	98.00%	-	-	-	-	Yes	Yes	-
(36)	Chanda et al. 2021	Zambia	100%	97.50%	-	100%	-	-	-	-	96.25%	-	97.50%	-	-	-	17.50%	10.00%	91.25%	-	-	-	-	-	93.75%	-	0.00%	5.00%	2.50%	Yes	-	Yes

Yes	-	-	-	-	-	-	-	-
-	-	-	Yes	-	-	-	-	-
-	-	Yes	-	Yes	-	-	-	-
-	-	-	-	2.80%	-	-	-	-
-	-	-	-	-	-	-	-	-
-	-	-	-	-	-	-	-	-
-	-	-	0.00%	2.70%	-	-	20.00%	-
-	-	97.50%	98.40%	97.70%	-	94.60%	91.10%	76%
-	-	1.9%	-	5.70%	-	-	1.40%	-
-	-	-	-	-	-	-	-	-
-	-	-	-	-	-	-	-	-
-	-	-	-	-	-	-	61.40%	-
-	-	-	-	72.10%	-	-	67.90%	83.25%
-	-	-	-	86.80%	-	82.00%	88.70%	44%
-	-	-	0.00%	-	-	0.00%	-	-
-	-	90.50%	94.00%	81.70%	-	53.50%	92.90%	98.50%
-	-	-	-	4.20%	-		42.20%	-
-	-	4.40%	0%	2.70%	-	7.40%	21.60%	-
-	-	-	-	-	-		99.40%	-
-	-	88.60%	99.40%	99.20%	-	99.50%	-	99.50%
-	-	-	-	-	-	66.20%	92.90%	-
39.00%	59.60%	76.60%	92.20%	50.80%	96%	81.90%	71.30%	98.00%
-	-	64.60%	75.00%	92.40%	-	55.60%	97.00%	93.50%
-	-	93.70%	-	2.70%	-	0.00%	-	-
-	-	19.30%	-	62.32%	-	0.90%	18.40%	24.10%
-	-	-	-	20.90%	-		29.00%	-
94.54%	-	94.90%	-	94.87%	-	74.00%	97.70%	-
-	-	-	-	-	-	-	-	-
-	100%	58.2%	88.00%	51.90%	-	80.20%	92.40%	-
-	100%	100%	99.60%	99.50%	-	98.20%	100%	-
Nigeria	Ghana	Nigeria	Ghana	Nigeria	South Africa	Sudan	Nigeria	Nigeria
Donald et al. 2021	Edzie et al. 2021	Garba et al. 2021	Jimah 2021	Robinson et al. 2021	Beviss-Challinor et al. 2020	A.Alameen et al. 2019	Chukwuma et al. 2019	Khadija et al. 2019
(37)	*(38)*	*(39)*	*(40)*	*(41)*	*(42)*	*(43)*	*(44)*	*(45)*

(46)	Mohammed et al. 2018	Nigeria	100%	95.10%	-	84.00%	42.90%	-	80.4%	72.40%	99.40%	-	-	100%	2.5%	-	-	-	-	-	-	-	-	-	-	-	-	-	-	-	-	Yes
(47)	Hk 2018	Ghana	99.00%	69.00%	-	-	-	-	-	61.00%	76.00%	-	98.00%	-	0.00%	-	86.00%	-	-	-	-	-	-	-	91.00%	0.00%	-	-	-	-	-	Yes
(48)	Onwuchekwa et al. 2017	Nigeria	100%	93.00%	-	82.00%	24.00%	41.00%	10.00%	79.00%	98.00%	-	100%	-	8.00%	8.00%	74.00%	2.00%	70.00%	77.00%	-	-	-	19.00%	97.00%	-	-	-	8.00%	-	Yes	Yes
(49)	Agi et al., 2015	Nigeria	100%	86.30%	-	97.30%	12.3%	-	-	-	91.7%	-	100%	-	-	-	91.00%	-	91.00%	81.30%	-	-	-	-	98.00%	-	-	-	-	-	-	-
(50)	Akintomide et al. 2015	Nigeria	97.40%	83.45%	-	95.30%	10.86%	11.50%	86.55%	86.55%	86.90%	71.55%	99.66%	-	69.00%	0.35%	-	-	85.86%	83.10%	-	-	-	-	-	-	-	-	-	Yes	-	Yes
(51)	Abubakar et al. 2015	Nigeria	100%	92.90%	-	95.90%	54.60%	2.10%	71.40%	77.30%	97.10%	-	98.50%		-	-	73.2%	-	94.40%	73.20%	-	-	-	12.40%	-	75.80%	-	-	-	Yes	-	-
(52)	Oyedeji et al. 2015	Nigeria	99.0%	86.0%	-	90.3%	5.6%	-	-	-	65.9%	-	-	-	-	-	99.0%	-	-	-	-	-	-	-	88.2%	-	-	-	-	-	Yes	-
(53)	Danfulani et al. 2015	Nigeria	100%	62.60%	-	100%	2.30%	-	100%	-	-	-	100%	-	-	-	2.30%	-	100%	-	-	-	-	-	100%	-	-	-	-	-	-	-
(54)	Moifo et al. 2014	Cameron	100%	74.00%	-	-	21.00%	-	-	-	82.80%	23.70%	63.00%	-	-	-	92.00%	4.20%	52.67%	-	-	-	3.90%	-	92.00%	-	-	-	-	-	-	-

-	Yes	-	-	-	-
-	Yes	-	Yes	-	Yes
-	Yes	-	Yes	Yes	-
-	-	-	-	-	-
-	-	-	-	-	-
-	-	-	-	-	-
-	-	-	-	-	-
97.00%	-	92.00%	-	-	98.60%
-	-	20.70%	-	-	1.40%
-	-	-	-	-	-
-	-	-	-	-	-
-	-	-	-	-	-
-	93.10%	99.70%	97.00%	88.70%	97.20%
-	96.50%	97.70%	75.60%	-	56.30%
-	-	-	-	-	0.0%
-	84.20%	98.70%	84.86%	88.30%	37.2%
-	59.80%	3.30%	-	-	14.6%
-	35.60%	-	10.57%	15.80%	11.10%
--	59.40%	-	-	-	93.10%
-	-	100%	82.85%	94.80%	100%
-	-	90.00%	-	-	41.00%
97.60%	64.90%	87.00%	20.00%	81.50%	18.20%
-	88.60%	98.30%	-	94.00%	92.40%
-	87.10%	92.30%	-	60.20%	-
-	-	-	7.10%	-	-
-	11.90%	13.00%	2.10%	29.70%	50.00%
-	96.00%	99.70%	-	98.80%	99.30%
-	-	-	-	-	
-	44.10%	98.00%	20.57%	79.30%	33.1%
-	89.10%	100%	100%	99.83%	98.60%
South Africa	Nigeria	Nigeria	Sudan	Nigeria	Nigeria
Schouwenburg et al. 2014	Afolabi et al. 2012	Irurhe et al. 2012	Yousef et al. 2011	Adebayo et al. 2009	Akinola et al. 2009
(55)	*(56)*	*(57)*	*(58)*	*(59)*	*(60)*

Table S 3 Issues highlighted by researchers

Reference	*Author, year of publication*	*Important highlighted issues*
(31)	Ademola et al. 2023	- More than half of the requests had inadequate clinical information, and lacking body part, and contact details of the referring doctor.<br>- No details of previous images and previous surgical history in 97% and 87% of the forms.<br>- 34% of the requested exams were inappropriate.<br>- LMP was not incorporated in the institutional form.
(32)	Bwalya et al. 2023	- 90% of the forms were incompletely filled.<br>- Clinical history was not provided in more than half of the form.<br>- The institutional RRF did not include LMP, past surgical and radiological history, clinician's phone number, consultant name.
(33)	Ahmed et al. 2022	- The bulk of inappropriate requests were for ionisation radiation modalities.<br>- Half of request did not select the appropriate medical imaging test particularly CTs for traumatic cases.<br>- LMP was poorly reported for females in the reproductive age group.
(34)	Chi et al. 2022	- A minority of RRFs were informal and excluded from the analysis.<br>- None of the requests was completely filled and only patient's name and location, and type of the exam were completely filled in all forms.<br>- None of the referring clinicians provide their contact number<br>- The patient's address was absent for nearly half of the forms.<br>- The majority of requests were devoid of LMP and allergy status.<br>- More than half of the requests did not provide a provisional diagnosis
(35)	Kuvare et al. 2022	- Nearly a third of the RRFs were unclear and illegible
(36)	Chanda et al. 2021	- Usage of informal forms.<br>- Patient's name and gender were the only fields filled in all forms<br>- Creatinine levels, allergic and diabetes statuses were absent in nearly all CT request forms
(37)	Donald et al. 2021	- Usage of informal RRF.<br>- Nearly half of the requests did not provide clinical information.

		- Sizable number of the request was inappropriate
(38)	Edzie et al. 2021	- Significant proportion without clinical information/indication.
(39)	Garba et al. 2021	- 47% of the exams were repeated because of inadequate clinical information.
(40)	Jimah 2021	- None of the requests were completely filled.<br>- Some vascular ultrasonography did not specify whether the arterial or venous exam is requested.<br>- More than half of the requests included inappropriate abbreviation.<br>- Twenty per cent of clinical history were useful or unavailable.<br>- Clinical question field was not incorporated into the institutional form.<br>- None of the referrers provide their contact details.
(41)	Robinson et al. 2021	- Clinical information were unavailable, incomplete or useful in half of the request.<br>- Nearly all of the requests did not state the allergic status to pharmaceutical agents.
(42)	Beviss-Challinor et al.. 2020	- None of the requests provide full clinical details
(43)	A.Alameen et al. 2019	- All RRF were incompletely filled.<br>- LMP was missed in 99.1% of the cases.<br>- Absence of referrer's details significantly correlated with clinical problem, clinical diagnosis, previous radiology, and ward, date of the request, gender and age of the patient.<br>- Referrer's contact number was missed in all forms.
(44)	Chukwuma et al. 2019	- Significant omission of patient's address, details of previous exams, LMP, and ambulatory status.
(45)	Khadija et al. 2019	- Significant omission of LMP.
(46)	Mohammed et al. 2018	- patient's address is omitted in more than half of the request.
(47)	Hk 2018	- fields of gender, DOB, hospital number, and referrer's signature, and contact number were not enlisted on the form.<br>- Usage of informal forms.

(48)	Onwuchekwa et al. 2017	- LMP was not enlisted in the request form.
(49)	Agi et al., 2015	- Large numbers of forms were poorly filled and only the patient's name and the requested exam was completely filled in all forms.
(50)	Akintomide et al. 2015	- The most completed request form was 86.6% filled and the majority of the form is 51% - 70%.<br>- Approximately a third of the forms was informal.<br>- 7% of the forms were illegible.<br>- Radiologists sometimes visit the wards to clarify and complete poorly completed information.
(51)	Abubakar et al. 2015	- LMP was the most common blank field with a completion rate of 2.1%.
(52)	Oyedeji et al. 2015	- Only 1.6% of 7841 requests were completely filled; including radiological, laboratory and cardiac investigations.
(53)	Danfulani et al. 2015	- 97.7% of the requests were inadequately filled.
(54)	Moifo et al. 2014	- Patient's name was the only field filled in all forms.<br>- In 30% of the forms, symptoms were the only stated clinical information.<br>- None of the requesting doctors provide their phone numbers.
(55)	Schouwenburg et al. 2014	- 2.4% of the requests were unjustifiable due to incomplete information.
(56)	Afolabi et al. 2012	- Patient's age was the most common incomplete field.
(57)	Irurhe et al. 2012	- Significant omission of patient's address and mobility state.
(58)	Yousef et al. 2011	- Some RRFs lacked standard fields such as LMP.<br>- All of the forms contain abbreviations and most of them were unstandard.
(59)	Adebayo et al. 2009	- Some forms were illegible and did not include the requested exam.<br>- Significant omission of patient's address, and number and details of previous exam,
(60)	Akinola et al. 2009	- Significant omission of patient's address and referrer's contact details.<br>- Most of the forms did not include adequate clinical information and provisional diagnosis.<br>- Non-universal abbreviations were used in all forms.
